# Leveraging the Electronic Medical Record in C. difficile Diagnostic Stewardship

**DOI:** 10.1017/ash.2024.186

**Published:** 2024-09-16

**Authors:** Vijay Duggirala, Jamaal Saleh, Justin Smyer, Courtney Hebert, Christina Liscynesky, Nora Colburn, Shandra Day

**Affiliations:** The Ohio State University Wexner Medical Center; The Ohio State University

## Abstract

**Background:** Clostridioides difficile PCR is extremely sensitive but cannot differentiate colonization versus active disease. Over diagnosis of C. difficile infection (CDI) has negative consequences including overuse of antibiotics targeting C. difficile, increased hospital-acquired (HA)-CDI rates, and increased healthcare costs. We describe the implementation of a Clinical Decision Support tool embedded in the C. difficile order and the result on testing, HA-CDI rates and healthcare costs. **Methods:** The C. difficile order was updated in June 2023 with 4 dynamic questions that reflex if specific criteria are identified in the electronic medical record in the prior 24 hours: less than 3 loose stools documented, receipt of laxative, opioid antagonist, oral contrast, or tube feed initiation. If any criteria are identified, an embedded alert triggers and the provider must choose “yes, high clinical suspicion” or “no (exit and cancel order)” in addition to providing an order indication. All inpatient C. difficile tests were reviewed from July 1 to Sept 30, 2022 (pre-update) and July 1 to Sept 30, 2023 (post-update). An order rate was calculated per 10,000 patient days as well as HA-CDI rate. Cost analysis was completed using direct lab costs and published costs of $35,000 per HA-CDI. Results of the order questions were reviewed post-update. Incident rate comparison was completed using medcalc. **Results:** Pre-update, 1147 tests were conducted, with an order rate of 104.3. Post-update, 919 tests were performed, with an order rate of 86.6. The positivity rate was 16.1% pre-update and 14.7% post-update. The incidence rate difference was 0.00177 (P 15 (145, 16%). 166 (18%) patients who received laxatives (18 positive, positivity rate 11%) were still tested. **Conclusion:** Implementation of a dynamic order led to a significant reduction in the total number of C. difficile PCR tests performed with associated reduction in HA-CDI and cost savings. Despite this, patients receiving laxatives were still being tested for C. difficile, highlighting the need for ongoing education and feedback. These results support the use of dynamic ordering for diagnostic stewardship, which can benefit both patients and hospitals.

